# Neutralization profiles of HIV-1 viruses from the VRC01 Antibody Mediated Prevention (AMP) trials

**DOI:** 10.1371/journal.ppat.1011469

**Published:** 2023-06-29

**Authors:** Nonhlanhla N. Mkhize, Anna E. J. Yssel, Haajira Kaldine, Rebecca T. van Dorsten, Amanda S. Woodward Davis, Nicolas Beaume, David Matten, Bronwen Lambson, Tandile Modise, Prudence Kgagudi, Talita York, Dylan H. Westfall, Elena E. Giorgi, Bette Korber, Colin Anthony, Rutendo E. Mapengo, Valerie Bekker, Elizabeth Domin, Amanda Eaton, Wenjie Deng, Allan DeCamp, Yunda Huang, Peter B. Gilbert, Asanda Gwashu-Nyangiwe, Ruwayhida Thebus, Nonkululeko Ndabambi, Dieter Mielke, Nyaradzo Mgodi, Shelly Karuna, Srilatha Edupuganti, Michael S. Seaman, Lawrence Corey, Myron S. Cohen, John Hural, M. Juliana McElrath, James I. Mullins, David Montefiori, Penny L. Moore, Carolyn Williamson, Lynn Morris

**Affiliations:** 1 National Institute for Communicable Diseases of the National Health Laboratory Service, Johannesburg, South Africa; 2 SA MRC Antibody Immunity Research Unit, Faculty of Health Sciences, University of the Witwatersrand, Johannesburg, South Africa; 3 Institute for Infectious Diseases and Molecular Medicine, Division of Medical Virology, Faculty of Health Sciences, University of Cape Town, Cape Town, South Africa; 4 South African Medical Research Council Antiviral Gene Therapy Research Unit, School of Pathology, Faculty of Health Sciences University of the Witwatersrand, Johannesburg, South Africa; 5 Vaccine and Infectious Disease Division, Fred Hutchinson Cancer Center, Seattle, Washington, United States of America; 6 Department of Microbiology, University of Washington, Seattle, Washington, United States of America; 7 Los Alamos National Laboratory, Los Alamos, New Mexico, United States of America; 8 Department of Surgery, Duke University, Durham, North Carolina, United States of America; 9 University of Zimbabwe College of Health Sciences Clinical Trials Research Centre, Harare, Zimbabwe; 10 Division of Infectious Diseases, Department of Medicine, Emory University, Decatur, Georgia, United States of America; 11 Center for Virology and Vaccine Research, Beth Israel Deaconess Medical Center, Harvard Medical School, Boston, Massachusetts, United States of America; 12 Department of Laboratory Medicine, University of Washington, Seattle, Washington, United States of America; 13 Department of Medicine, University of North Carolina at Chapel Hill, Chapel Hill, North-Carolina, United States of America; 14 Centre for the AIDS Programme of Research in South Africa (CAPRISA), University of KwaZulu Natal, Durban, South Africa; 15 National Health Laboratory Service, Cape Town, South Africa; University of Illinois at Chicago College of Medicine, UNITED STATES

## Abstract

The VRC01 Antibody Mediated Prevention (AMP) efficacy trials conducted between 2016 and 2020 showed for the first time that passively administered broadly neutralizing antibodies (bnAbs) could prevent HIV-1 acquisition against bnAb-sensitive viruses. HIV-1 viruses isolated from AMP participants who acquired infection during the study in the sub-Saharan African (HVTN 703/HPTN 081) and the Americas/European (HVTN 704/HPTN 085) trials represent a panel of currently circulating strains of HIV-1 and offer a unique opportunity to investigate the sensitivity of the virus to broadly neutralizing antibodies (bnAbs) being considered for clinical development. Pseudoviruses were constructed using envelope sequences from 218 individuals. The majority of viruses identified were clade B and C; with clades A, D, F and G and recombinants AC and BF detected at lower frequencies. We tested eight bnAbs in clinical development (VRC01, VRC07-523LS, 3BNC117, CAP256.25, PGDM1400, PGT121, 10–1074 and 10E8v4) for neutralization against all AMP placebo viruses (n = 76). Compared to older clade C viruses (1998–2010), the HVTN703/HPTN081 clade C viruses showed increased resistance to VRC07-523LS and CAP256.25. At a concentration of 1μg/ml (IC80), predictive modeling identified the triple combination of V3/V2-glycan/CD4bs-targeting bnAbs (10-1074/PGDM1400/VRC07-523LS) as the best against clade C viruses and a combination of MPER/V3/CD4bs-targeting bnAbs (10E8v4/10-1074/VRC07-523LS) as the best against clade B viruses, due to low coverage of V2-glycan directed bnAbs against clade B viruses. Overall, the AMP placebo viruses represent a valuable resource for defining the sensitivity of contemporaneous circulating viral strains to bnAbs and highlight the need to update reference panels regularly. Our data also suggests that combining bnAbs in passive immunization trials would improve coverage of global viruses.

## Author summary

HIV-1 viruses from the Antibody Mediated Prevention (AMP) trials, obtained in 2016–2020, represent our most recent panel of transmitted viruses in the population. Reference virus strains that are currently used to evaluate broadly neutralizing antibodies (bnAb) for passive immune trials and assess the quality of vaccine induced antibody responses, were collected between 1998 and 2010. As HIV-1 continues to evolve, virus panels may need to be updated from time to time. Sequences were obtained from participants infected with HIV-1 from both the African (HVTN 703/HPTN081) and the Americas/European (HVTN 704/HPTN085) AMP trials. These viruses were then tested in a neutralization assay with 8 bnAbs under clinical development. Comparing the neutralization coverage with older viruses, we found that recent viruses have become more resistant to VRC07-523LS and CAP256.25 antibodies. Predictive modelling identified triple bnAb combinations that would be effective at neutralizing HIV-1 strains from different geographical regions. Reference panels that include recently transmitted HIV-1 strains provide important information that can be used to design bnAb combinations to consider for prevention studies.

## Introduction

Broadly neutralizing antibodies (bnAbs) represent a promising approach for the prevention of human immunodeficiency virus (HIV-1) infection. Several studies have demonstrated that passively administered antibodies can protect non-human primates (NHP) from Simian-human immunodeficiency virus (SHIV) infection [1–4], however this had not been assessed in humans until recently. Through a large, coordinated effort, the Antibody Mediated Prevention (AMP) trials evaluated whether long-term administration of a passively infused bnAb VRC01 (which targets the CD4 binding site, CD4bs) could prevent HIV-1 acquisition in humans [5]. A total of 4,600 at-risk participants were enrolled in the AMP trial from diverse geographical regions including countries in Sub-Saharan Africa (South Africa, Zimbabwe, Botswana, Tanzania, Kenya, Malawi, Zambia), America (USA, Peru, Brazil), and Europe (Switzerland). The results demonstrated that VRC01 could prevent HIV-1 infection, but that prevention efficacy was dependent on the sensitivity of circulating viruses to VRC01 [5]. Thus, prevention efficacy was high (75%) against viruses that were sensitive to VRC01 (IC80 <1μg/ml) but there was no efficacy against viruses with IC80 values >1μg/ml which constitute the majority of circulating strains and no significant protection overall.

The viruses isolated early in infection during the AMP trials provide a valuable tool going forward. There is evidence that over time, circulating viruses have diversified significantly and become more resistant to certain, previously isolated bnAbs [6,7]. A study of 200 Clade C viruses collected early in infection between 1998–2010 showed an increase in neutralization resistance over time to antibodies targeting the CD4bs (VRC01), V2-glycan (PG9) and MPER (4E10) [6]. This evolution resulted in a near doubling of the VRC01 concentration needed to reach 50% inhibition of clade C viruses over these 13 years. Clade B viruses have exhibited the same pattern of resistant phenotypes emerging over time [7,8]. Resistance to neutralization has been attributed to amino acid changes, V1V2 loop length, and additions of glycans [6,9–11]. These observations suggest that viral reference panels need to be updated periodically to obtain accurate sensitivity data for new bnAbs under investigation. Several neutralization reference panels exist and comprise viruses that have been characterized and tested against different sera and bnAbs [6,12,13]. However, these panels represent viruses circulating between 1998 and 2010, highlighting the urgent need to characterize the contemporaneous circulating viruses provided by the AMP trials a decade later.

The AMP trial also emphasized the fact that future bnAb trials will require combinations of more potent antibodies with better coverage to improve overall prevention efficacy. Many Phase I trials utilizing more potent and/or broader bnAbs are underway, including assessments of combinations of bnAbs in HIV-1 prevention and suppression [14,15]. Predictive statistical modeling has suggested that second-generation bnAbs, particularly when used in combination, can neutralize close to 100% of global HIV-1 strains [16,17]. However, these bnAb coverage predictions are only accurate if modeled using data generated with currently circulating viruses.

Here, we show the sensitivity of viruses from AMP trial placebo recipients isolated early during infection (within 45 days from the estimated time of acquisition) to clinically relevant bnAbs and identify signatures that may be responsible for genetic drift. Our study provides data that will be useful to evaluate bnAb candidates for passive immunization trials and also offers an opportunity to update reference panels.

## Results

### Phylogenetic sequence analysis of viruses acquired by AMP trial participants

**Fig 1:**
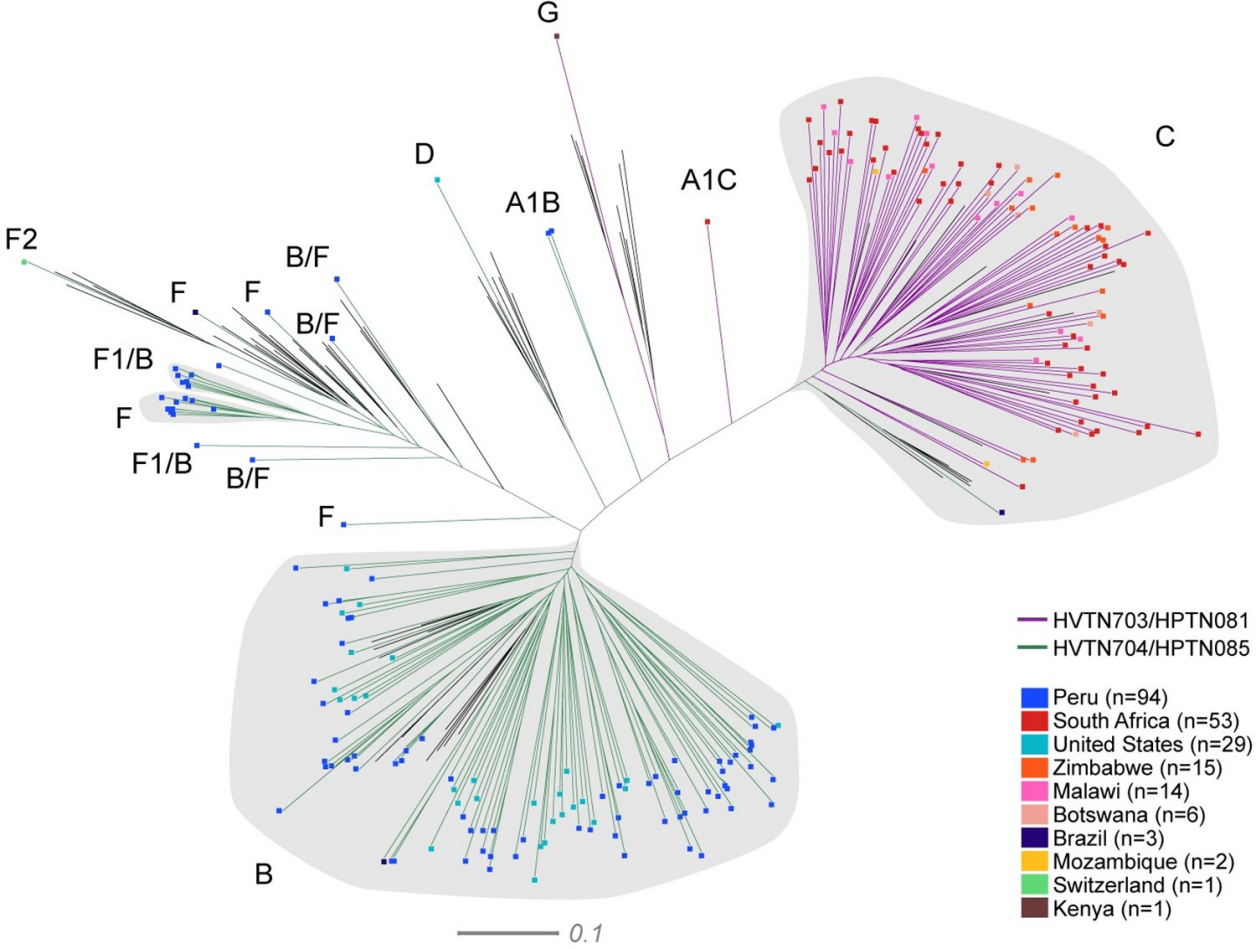
Phylogenetic analysis of envelope nucleotide sequences from the AMP trials. Sequences from all arms of HVTN703/HPTN081 (purple branches) and HVTN704/HPTN085 (green branches) are shown in the tree. Each participant is represented by a single Env gene sequence. The ends of the branches are coloured according to the country of origin: Peru (bright blue), South Africa (red), United States (teal), Zimbabwe (orange), Malawi (pink), Botswana (dark teal), Brazil (dark blue), Mozambique (yellow), Switzerland (blue), and Kenya (brown). Branches in black (with no coloured ends) represent reference strains from different clades. The scale is shown at the bottom.

HIV-1 Env were sequenced from 218 infections in both AMP trials. In some cases, more than one Env was sequenced from an infection and the dominant clone was selected for analysis. There were 91 HIV-1 infections across all the study arms of the HVTN 703/HPTN 081 AMP trial (placebo = 33, low dose = 36, high dose = 22), with 89 participants infected with clade C, one with clade G (from Kenya) and one with an A/C recombinant (South Africa) (**Fig 1**). Most infections were from South Africa (n = 53), followed by Zimbabwe (n = 15), Malawi (n = 14), Botswana (n = 6), Mozambique (n = 2) and Kenya (n = 1). There were no infections from the Tanzania site at the time of our analysis. Phylogenetic analysis of the envelope sequences from HVTN 703/HPTN 081 demonstrated an equal spread of clade C sequences across the tree regardless of country of origin.

In the HVTN 704/HPTN 085 trial, there were 127 HIV-1 infections across all arms (placebo = 47, low dose = 42, high dose = 38). There was a higher clade diversity of HIV-1 envelope sequences in this trial compared to HVTN 703/HPTN 081 (**Fig 1**): 94 participants acquired clade B, 14 clade F, 2 A1/B recombinants, 15 B/F recombinants, 1 B/D recombinant, 1 clade C, and 1 clade D subtypes. A large proportion of infections were from Peru (n = 94), and the United States of America (n = 29) followed by Brazil (n = 3) and Switzerland (n = 1). Clade B sequences in HVTN 704/HPTN 085 did not cluster by country of origin, however the majority of F and B/F recombinants were identified in South American participants.

### Neutralization sensitivity of AMP pseudoviruses to clinically relevant bnAbs

**Fig 2:**
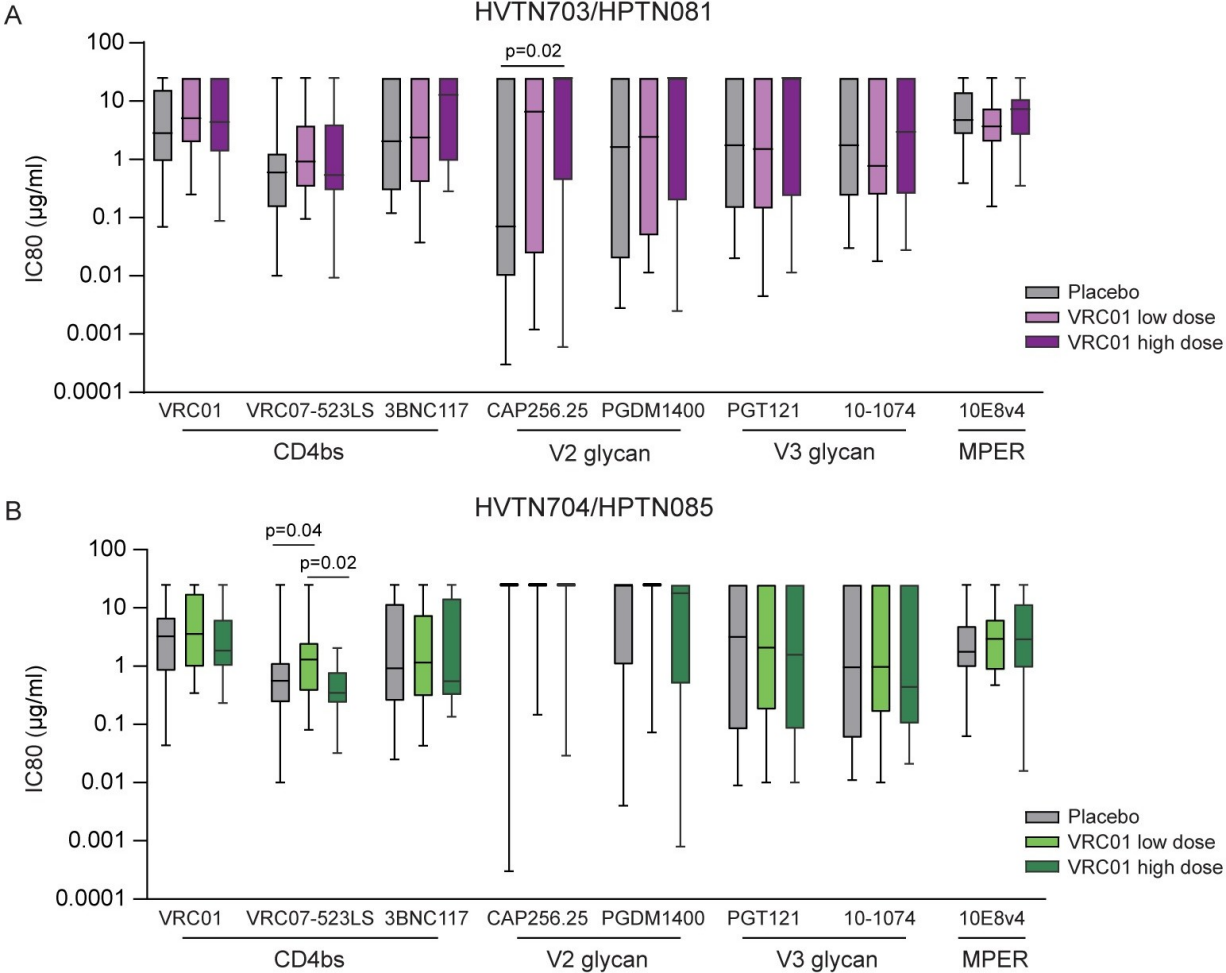
Neutralization profiles of pseudoviruses from the different study arms of the AMP trials. Box plots illustrate neutralization data (IC80 titers, median and range) of all viruses against 8 bnAbs in clinical development and separated according to study arms in **(A)** HVTN703/HPTN081 (placebo n = 33, low dose n = 35, high dose n = 21) and **(B)** HVTN704/HPTN085 (placebo n = 47, low dose n = 42, high dose n = 38). Solid bars represent median titers.

An assessment of VRC01 neutralization sensitivity using pseudoviruses generated from the envelope sequences was part of the primary analysis of the trial [5], which showed that less than 30% of these pseudoviruses were sensitive to VRC01 at an IC80< 1μg/ml. In this study, we further examined their sensitivity to seven bnAbs undergoing clinical testing, representative of four distinct epitope regions: CD4bs (VRC07-523LS, 3BNC117), V2 glycan (CAP256.25, PGDM1400), V3 glycan (PGT121, 10–1074), and MPER (10e8v4). An analysis of the median neutralization titers showed VRC07-523LS as the most potent (IC80<1μg/ml) CD4bs bnAb against pseudoviruses in the placebo and high dose groups of HVTN 703/HPTN 081 and HVTN 704/HPTN 085 (**Fig 2A and 2B**). The IC80 neutralization sensitivity for all 1,248 env-pseudovirus/bnAb combinations, which includes all 317 Envs sequenced from 218 infections, is shown in heat maps (**S1 Table**). There was a significant difference between the pseudoviruses in the placebo and the low dose (p = 0.04) and high dose (p = 0.02) arms for VRC07-523LS but not with the other bnAbs in HVTN 704/HPTN 085 (**Fig 2B**). A similar trend was observed for VRC01 in both trials, although this was not significant.

### Neutralization profiles of AMP placebo viruses

**Fig 3:**
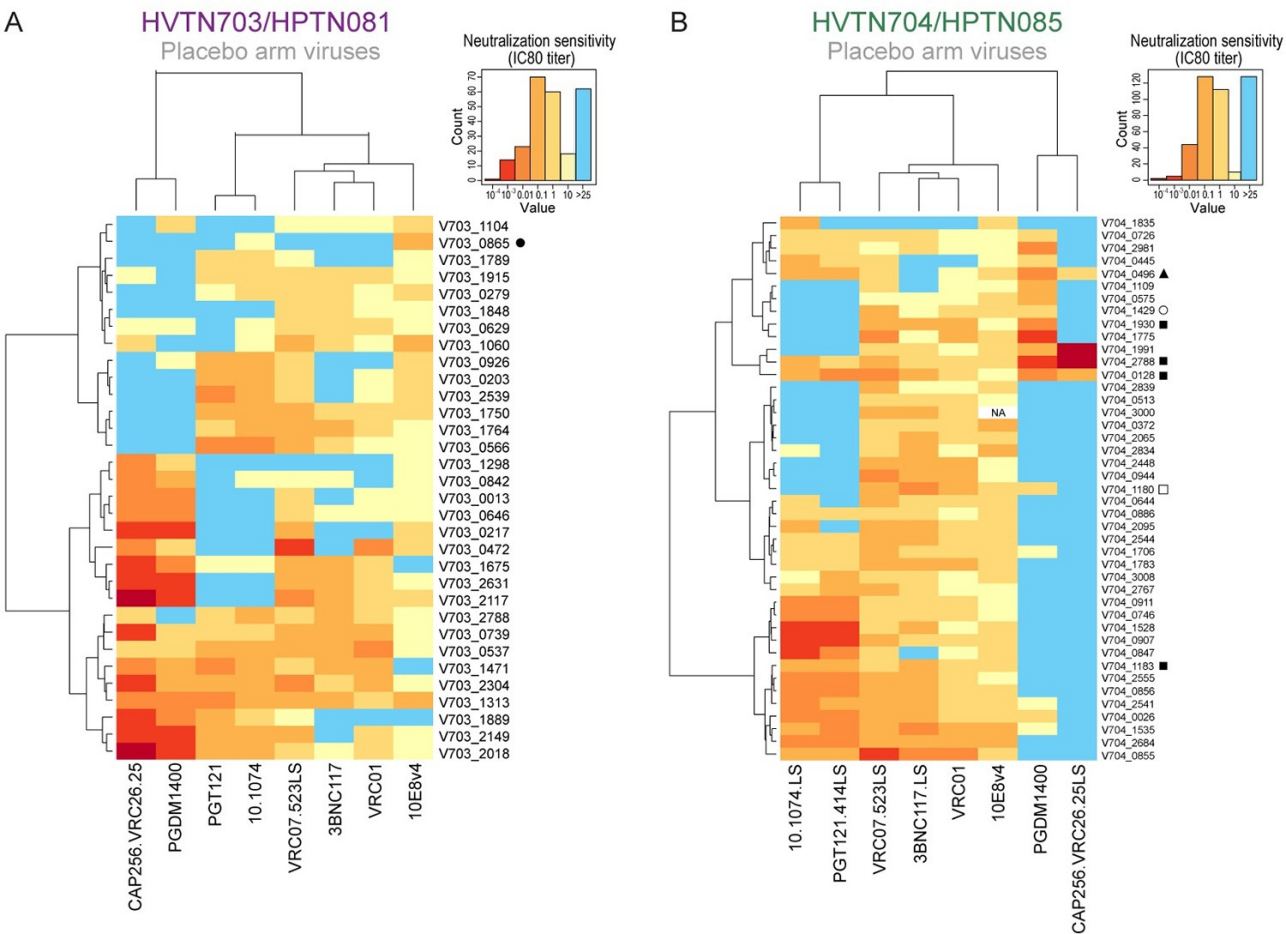
Neutralization profiles of pseudoviruses from the placebo arms of the AMP trials. Hierarchical clustering was utilized to analyze virus/bnAb combinations for HVTN703/HPTN081 (**A**) and HVTN704/HPTN085 (**B**) viruses were clustered according to their neutralization profiles along the vertical axes and bnAbs by sensitivity along the horizontal axes. A tally of the number of viruses by neutralization sensitivity (IC80 titer) and colour key is shown on the upper right corners of the plots. All sequences for HVTN703/HPTN081 are clade C and all sequences for HVTN704/HPTN085 are clade B unless otherwise denoted (●G, ▲F, ○F2, □ A/B recombinant, ■B/F recombinant).

Next, we focused on the pseudoviruses in the placebo arms (n = 76) for both AMP trials (hereafter referred to as “AMP placebo viruses”) and generated a hierarchical clustering heatmap comparing viral neutralization against the same bnAbs (**Fig 3**). We did not include viruses in the VRC01 arm in this analysis as antibody pressure may have led to selection [18]. The greatest breadth in HVTN 703/HPTN 081 was seen with VRC07-523LS (**Fig 3A**) and 10E8v4 neutralized all the viruses tested in HVTN 704/HPTN 085 (**Fig 3B**). There was a clear grouping between V2-glycan bnAb sensitive and non-sensitive viruses in HVTN 703/HPTN 081 with a clear overlap in sensitivity except for 5 viruses that were differentially sensitive to either CAP256.25 or PGDM1400. Unlike in clade C-dominant HVTN 703/HPTN 081, HVTN 704/HPTN 085 had a low frequency of viruses sensitive to CAP256.25 (4/43) and PGDM1400 (17/43) (**Fig 3B**). This is consistent with previous results showing that clade B viruses are not well neutralized by V2-glycan targeting antibodies [19–21].

### Comparison of clade C AMP placebo arm viruses to historical Clade C Panel viruses

**Fig 4:**
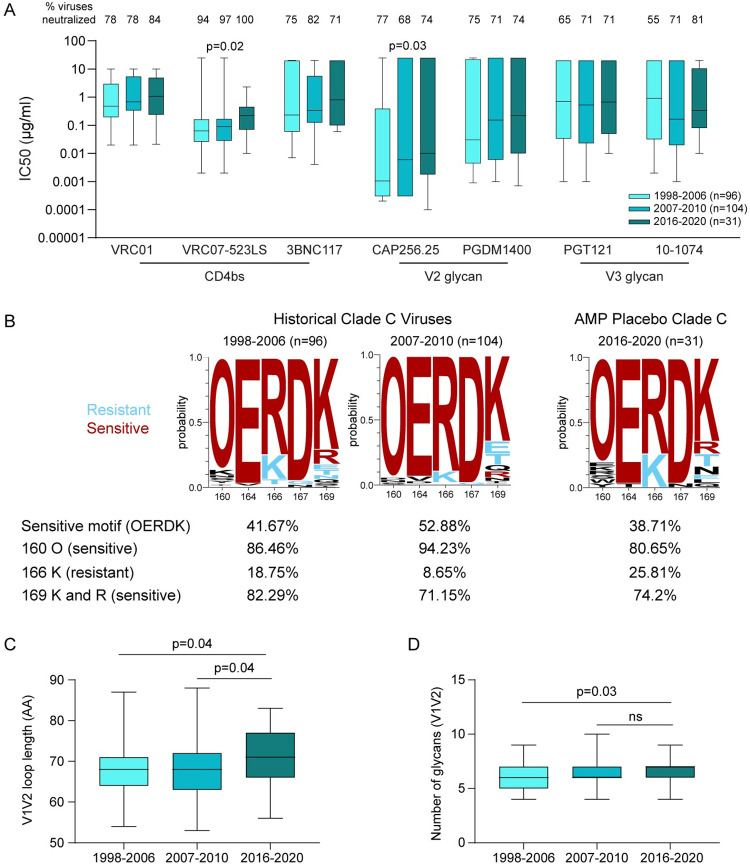
Neutralization sensitivity and genetic characteristics of historical clade C viruses compared to AMP placebo viruses. **(A)** Previously characterized clade C panel viruses were analyzed according to time of collection (1998–2006, 2007–2010) and their sensitivity to bnAbs was compared to placebo viruses from AMP (2016–2020). The cut-off IC50 for VRC01 is 10μg/ml; 20μg/ml for 3BNC117, PGT121 and 10–1074; 25μg/ml for VRC07-523LS, CAP256.25 and PGDM1400. The Jonckheere-Terpstra trend test was used to determine changes in neutralization sensitivity over time and p<0.05 was considered significant. **(B)** logogram of clade C viruses for sites linked to CAP256.25 resistance/sensitivity for the three time periods. The amino acid frequency (y-axis) is shown for each residue at positions 160,164,166,167 and 169. The size of each AA in the logogram is proportional to its frequency and the “O” represents N-linked glycans. Residues associated with a sensitive neutralization phenotype are in red while residues in blue represent resistant residues. Residues that are not associated with either resistant or sensitive are in black. **(C)** V1V2 loop length and **(D)** the number of potential N-linked glycosylation sites were compared across the three time periods. The Mann-Whitney test was used to compare differences between the groups and p<0.05 was considered significant.

We and others have previously shown that antigenic drift impacts bnAb sensitivity [6–8]. We therefore compared clade C viruses from the placebo arm of the HVTN 703/HPTN 081 AMP trial (n = 31) to the previously characterized 200 Clade C Panel of viruses collected between 1998 and 2010. These historical viruses were split into two roughly equal groups: 1998–2006 (n = 96) and 2007–2010 (n = 104). All viruses were assayed against the same eight bnAbs targeting four distinct epitopes. The AMP placebo viruses were found to be significantly more resistant to the CD4bs bnAb VRC07-523LS (p = 0.02) and the V2-glycan specific bnAb CAP256.25 (p = 0.03) compared to historical viruses (**Fig 4A**). A trend towards increased resistance to VRC01, 3BNC117 and PGDM1400 was also observed over time although significance was not reached. PGT121 and 10–1074 neutralization sensitivity remained unchanged over the periods assessed.

### Diversity of HIV-1 sequences in the V2 bnAb epitope

Since we had observed a significant change in the sensitivity of viruses to CAP256.25 over time, we next evaluated the amino acid (AA) changes in key contact and signature sites in viruses across the 3 time periods (1998–2006, 2007–2010, 2016–2020) [22] (**Fig 4B**). The AAs that make up the sequence “OERDK” are each associated with sensitivity to CAP256.25 [22,23] and we identified a decrease in the frequency of viruses with this motif in the recent time interval. There was an overall decrease in frequency of sites associated with sensitivity to CAP256.25 (the glycan at position 160, the Lysine and Arginine at 169) and an increase in frequency of sites associated with resistance to CAP256.25 (Lysine at 166). We next investigated whether V1V2 loop lengths and glycan density changed over time as these features are linked to neutralization sensitivity. Current viruses (2016–2020) had significantly longer V1V2 loops compared to the older viruses (**Fig 4C**). Glycan density in the V1V2 region was also significantly higher in the current viruses compared to viruses from the earliest group (1998–2006) (**Fig 4D**). Overall, this result supports the more resistant neutralization phenotype of newer viruses against CAP256.25 (**Fig 4B**).

### Predicted coverage of triple bnAb combination against the AMP placebo viruses

**Fig 5:**
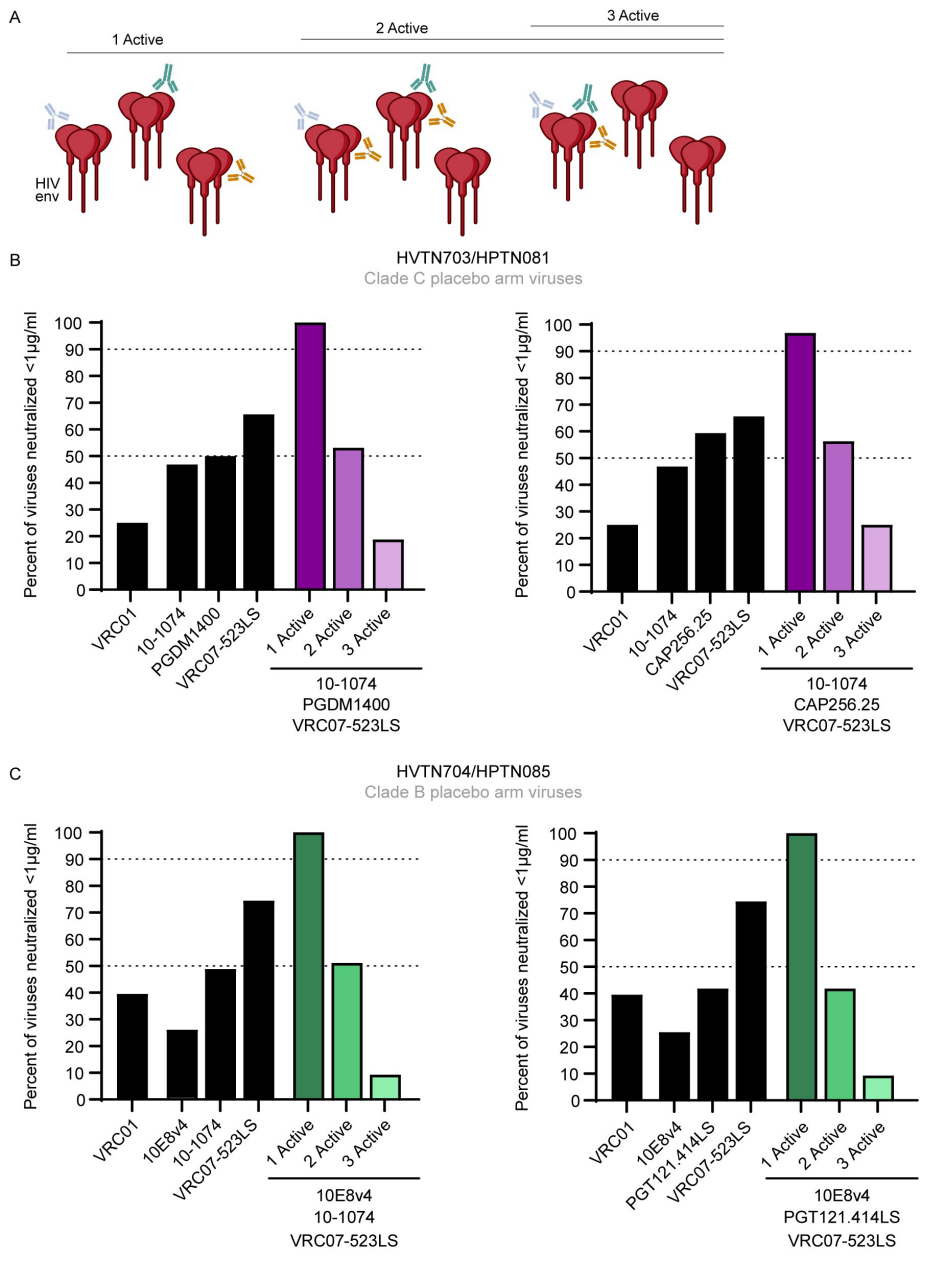
Predicted coverage of bnAb combinations against circulating viruses. **(A)** Diagram illustrating neutralization coverage (created with BioRender.com). “1 Active” is the percent of viruses neutralized by at least one bnAb, “2 Active” is the percent of viruses that can be neutralized by at least two bnAbs, “3 Active” is the percent of viruses that can be neutralized by all three bnAbs. Neutralization coverage by single antibodies (black) was calculated at IC80 <1μg/ml.Using the Bliss Hill Independence model, the active coverage of the antibodies in triple combinations (purple shades for HVTN703/HPTN081 and green shades for HVTN704/HPTN085) was calculated based on the single antibody neutralization data with **(B)** HVTN703/HPTN081 (clade C) viruses and **(C)** HVTN704/HPTN085 (clade B) viruses. Coverage at 50% and 90% is indicated by the dotted lines.

The AMP trial demonstrated that viruses that were sensitive to VRC01 with an IC80 of <1μg/ml were blocked from establishing infection [5]. Therefore, we used the neutralization data we generated from the AMP placebo viruses to predict active coverage, illustrated schematically in (**Fig 5A**) at IC80 <1μg/ml and potent and broad bnAb combinations. We focused on clade C viruses from HVTN 703/HPTN 081 and clade B viruses from HVTN 704/HPTN 085 (**Fig 5B and 5C**).

Clade C coverage by individual antibodies was less than 50% for all bnAbs (IC80 <1μg/ml) except for CAP256.25 (~59%) and VRC07-523LS (~70%) (**Fig 5B and S1 Table**). Combinations of three bnAbs, where at least one of them can neutralize the virus, increased coverage to close to 100% as expected (**Fig 5**). The best triple combinations against clade C viruses were 10-1074/PGDM1400/VRC07-523LS which resulted in 100% coverage and 10-1074/CAP256.25/VRC07-523LS with 97% coverage (**Fig 5B and S2 Table**). However, this coverage was reduced to around 50% in a dual active scenario (the proportion of viruses neutralized by at least two bnAbs) and around 20% when modeling triple active coverage (the proportion of viruses neutralized by all three bnAbs). Replacing PGDM1400 with CAP256.25 decreased single coverage slightly but achieved higher dual and triple active coverage.

Predictive modeling of clade B viruses identified 10E8v4/10-1074/VRC07-523LS and 10E8v4/PGT121.414LS/VRC07-523LS as the best triple combinations with coverage of 100% for both (**Fig 5C and S2 Table**). Replacing 10–1074 with PGT121.414LS decreased the dual active coverage from 52% to 39% but the triple active coverage was maintained at 12%. Interestingly, substituting the V3 bnAbs (either 10–1074 and PGT121) with PGDM1400 still maintained a coverage of 100% for 1 active bnAb but the dual and triple-active coverage was reduced to 18% and 0% respectively.

## Discussion

Characterization of viruses from early in infection from both sub-Saharan African and American/European populations provides an important resource for assessing the potential efficacy of bnAbs under clinical development. Evolution of HIV-1 at a population level has been linked to the emergence of neutralization-resistant phenotypes [7,8] presenting a serious challenge to successful bnAb mediated prevention. Here we investigated viruses from the AMP trials which are representative of the HIV-1 epidemic across 11 countries in sub-Saharan Africa, North and South America and Europe between 2016 and 2020, offering relevant information about circulating viruses. Furthermore, these viral strains offer the opportunity to update previously established panels assembled with viruses between 1998 and 2010 [6,12,13], a necessary step towards HIV prevention and cure.

A considerable challenge facing the field is the continued evolution of HIV-1 resulting in evasion of immune responses. Reference panels have been utilized to standardize the comparison of breadth and magnitude of neutralization responses between trials [6,12,13]. The established 200 virus Clade C Panel being used for current studies is made up of viruses isolated between 1998 to 2010. Here, we collected clade C viruses isolated between 2016 and 2020 as a first step towards building an updated reference panel. Importantly, these recent viruses displayed increased neutralization resistance to CAP256.25 (p = 0.03) and VRC07-523LS (p = 0.02) compared to the historical 200 Clade C Panel. These results are consistent with data from Indian Clade C viruses showing resistance to CAP256.25 at a population level over time [24]. Rademeyer et al also observed that viruses became increasingly resistant to VRC01 (CD4bs), PG9 (V2-binding) and 4E10 (MPER/gp41-binding) over a 13-year period [6]. While a significant decrease in sensitivity to VRC01 was not observed in our study, we did note a trend towards resistance to VRC01 and 3BNC117, which share some signature contact sites [25–28], suggesting a shift in general resistance to CD4bs antibodies over time [6,24]. The increased resistance to CD4bs and V2 bnAbs suggests the possibility that similar responses are common in HIV infection and are responsible for applying selective pressure to viral populations. These data also have implications for active vaccination. For example, while promising proof-of-concept results with a vaccine able to elicit CD4bs bnAb precursors have recently been published [29], our study suggests that these VRC01-like bnAbs could show reduced activity against current viruses, further highlighting the need to evaluate the efficacy of new bnAbs with contemporaneous viruses.

A few specific hallmarks were apparent that could account for the increased resistance. Compared to the older 200 Clade C Panel viruses, recent viruses demonstrated an overall increase in glycans associated with resistance and decrease in glycans associated with sensitivity to CAP256.25 [22,23]. Furthermore, recent viruses had longer V1V2 loop lengths. Over the course of infection, it has been shown that viruses develop increased V1V2 loop lengths [30], thus affecting neutralization sensitivity [11,21,31,32]. Longer V1V2 loops may alter the conformation of the viral protein structure and affect sensitivity of the virus to bnAbs targeting different epitopes [11,33,34]. Our data supports these findings and emphasize the importance of taking these viral changes into consideration when evaluating candidate bnAbs.

The AMP trials demonstrated that HIV-1 prevention can be achieved by bnAb administration if the circulating viruses have a neutralization sensitivity (IC80) of <1μg/ml [5]. In our study comparing bnAbs of clinical interest, VRC07-523LS had the highest coverage (>70% at <1μg/ml) and potency against clade C viruses, consistent with the 200 Clade C Panel data [16], and also showed good coverage against clade B viruses. CAP256.25 potency was higher than that of VRC07-523LS when looking at the geometric mean titer of the sensitive viruses but had low breadth with ~60% coverage at IC80 <1μg/ml for clade C viruses and only <10% for clade B viruses. This indicates that in passive immunization trials, CAP256.25 would need to be administered in combination with a broader bnAb to increase the coverage of clade C viruses neutralized and would not be useful against clade B viruses.

We utilized predictive modeling to investigate the effectiveness of bnAb combinations against historical and currently circulating viruses in vitro. Phenotypic differences between clade C viruses from the African AMP trial and clade B viruses from the American/European AMP trial were reflected in the predicted bnAb combinations that would be the most effective. In our study, 10-1074/PGDM1400/VRC07-523LS was predicted to be the optimal combination achieving 100% coverage at IC80 = 1μg/ml against clade C viruses. Previously, Wagh et al identified the triple bnAb combination CAP256.25/VRC07-523/10-1074 as the best in terms of coverage and potency in a predictive model when using the 200 Clade C Panel viruses and bnAb data [16]. While both combinations include V2-glycan-targeting antibodies, we observed that the combination with CAP256.25 achieved 97% coverage. However, the dual-active and triple-active bnAb coverage with CAP256.25 performed slightly better than PGDM1400 against the recent viruses, possibly due to better complementarity of CAP256.25 with PGT121 at IC80<1μg/ml. Overall, selecting a triple combination that offers >90% coverage for 1 active antibody and at least 50% for dual active (different epitopes) may be beneficial to mitigate the effect of antibody escape.

While the best triple combinations against clade C viruses included V2/V3/CD4bs bnAbs, clade B viruses were better targeted by bnAbs that recognized MPER/V3/CD4bs, as we observed that 10E8v4/10-1074/VRC07-523LS and 10E8v4/PGT121.414LS/VRC07-523LS were the combinations with the highest coverage. Combinations with PGDM1400 had reduced dual and triple bnAb activity against clade B viruses due to the low coverage by V2-targeting antibodies, whereas 10E8v4, which has better overall breadth but lower potency, proved to be beneficial and achieved 100% coverage. Unfortunately, Phase 1 trials that were planned with 10E8 co-administered with other more potent antibodies were paused due to reactogenicity observed in a participant after infusion (NCT03565315). Efforts continue to engineer a safer, increased half-life version of 10E8 which is a promising candidate for bnAb combinations [35–37]. Combinations of potent antibodies for prevention will improve overall breadth and lessen the effect of antibody resistance/escape.

Several approaches are being utilized to address the limitations of breadth and neutralizing potency of current bnAbs, as highlighted by the AMP trial results [5]. Optimization of existing HIV-1 bnAbs for enhanced potency has generated potent bnAbs such as VRC01.23LS, achieving ~10-fold improved potency and breadth higher than the parental VRC01 antibody [38]. Similarly, VRC07 was engineered with a series of mutations to generate VRC07-523, which is also about 10-fold more potent than the parental bnAb [39]. Furthermore, bi-or tri-specific antibodies that combine Fab portions from different antibodies demonstrate enhanced breadth and potency compared to the single antibodies [40]. In addition to the above Fab changes, modifications to the Fc portion of neutralizing antibodies to improve the half-life in serum is crucial for the maintenance of therapeutic concentrations, therefore increasing prevention efficacy. The “LS” mutation engineered into bnAb clinical candidates extends longevity of circulating bnAbs [36,37]. For VRC01, the LS mutation increased the half-life fourfold [41]. Overall, the combination of improved neutralization potency, breadth, and extended half-life may generate antibodies that require lower doses and with longer boosting intervals, ultimately reducing clinical trial and treatment costs. Reduction in costs could bode well for the use of bnAbs as PrEP, but there still remains a challenge of resistance to bnAbs that may develop in individuals over time. Therefore, bnAbs would likely be adjunctive to existing PrEP regimens.

While this study had a limited sample size of current viruses (n = 31) being compared to the historical 200 Clade C Panel, there are emerging opportunities to expand our 2016–2020 virus repository to include viruses from other HVTN trials such as HVTN 702 [42] (NCT02968849) and HVTN 705 (NCT03060629). Despite the moderate neutralization sensitivity shifts of the clade C viruses in the last 10 years, HIV-1 continues to evolve and there is a need to replace older viral sequences (<2010 period) with currently circulating viral sequences in standard reference panels. In addition, most of the bnAbs in clinical development, and tested in this study, were isolated almost 10 years ago [43–45]. This time-lag may affect the sensitivity of current viruses to these bNAbs, and therefore, isolation and testing of bNAbs from more recent infections against contemporaneous virus panels will be of interest. Having relevant HIV-1 sequences for reference will ensure that an accurate evaluation of the effectiveness of bnAbs being tested is conducted, informing improved clinical trial design and ongoing development of superior bnAbs.

## Materials and methods

### Ethical statement

All work described here complied with all relevant ethical regulations. This work was approved by the Duke University Health System Institutional Review Board (Duke University) through protocol no. Pro00093087. For the National Institute for Communicable Diseases (NICD), the work was approved by the University of the Witwatersrand Human Research Ethics Committee through protocol no. M201105. The Institutional Review Boards/Ethic Committees of participating clinical research sites (CRS)approved the studies, which were conducted under the oversight of the NIAID Data Safety Monitoring Board (DSMB). All participants gave written informed consent.

### Study participants

The HVTN 703/HPTN 081 AMP trial enrolled 1,924 women at risk of HIV-1 infection from sub-Saharan Africa; 11 sites in South Africa, 3 in Zimbabwe, 2 in Malawi, and 1 each in Botswana, Kenya, Mozambique, and Tanzania. The HVTN 704/HPTN 085 AMP trial enrolled 2,704 men and transgender persons at risk of HIV-1 infection from the Americas and Europe; 19 sites in the United States, 5 in Peru, 1 in Brazil and 1 in Switzerland [5]. Participants were randomly assigned in a 1:1:1 ratio to receive placebo, intravenous VRC01 of 10 mg/kg (low dose), or 30 mg/kg (high dose) at 8-week intervals for 72 weeks. HIV-1 infection was determined by plasma RNA testing every 4 weeks.

### Sequencing of viruses from participants infected with HIV-1 in the AMP trials

Viruses from the first HIV-1 RNA positive visit were sequenced using Sanger consensus and single genome amplification and sequencing and/or PacBio Single-Molecule-Real Time (SMRT) sequencing [46]. The envelope sequence representing the major lineage was synthesized. Variants present in more than ~10% of the population were also synthesized resulting in 317 synthesized Env sequences from a total of 218 HIV-1 breakthrough infections.

### Pseudovirus production and neutralization assays

Env-pseudotyped viruses were produced via transfection of the envelope plasmids in 293T cells as previously described [47,48]. Neutralization sensitivity of the viruses to a panel of monoclonal antibodies was assessed in the TZM-bl neutralization assay [49,50] and compared to historical data (CATNAP *https*:*//www*.*hiv*.*lanl*.*gov*) [6]. Results were expressed as IC80 values which is the 80% inhibitory antibody concentration required to neutralize 80% of HIV-1 infection in TZM.bl target cells.

### Generation of phylogenetic trees

For subtyping, the env nucleotide sequence that was present at the highest frequency was selected per participant and was compared to a representative panel of clades (www.lanl.gov). An alignment was created with MACSE v2.06. An ML tree was generated with RAxML-ng (version 1.1.0) using the optimal model (GTR+I+G4, with ModelTest-ng), and 1000 bootstrap replicates. The tree was drawn and coloured with Dendroscope 3.7.6, and additional annotations added with InkScape 1.1.2. Trees illustrating neutralization sensitivities were generated using all synthesized env sequences and computed using FastTree2 [51] with a GTR+CAT model and drawn using the package ggtree [52].

### Bliss-Hill Independence model for predicting bnAb combination neutralization coverage

The IC80 values from bnAb combinations based on the Bliss-Hill Independence model were calculated as previously described [16,53].

$$f(c)=\frac{c^{m}}{\left(k^{m}+c^{m}\right)}$$

Where c = bnAb concentration, k = IC_50_, and

$$m=\frac{\log\left(4\right)}{\log\left(IC_{80}\right)-\log\left(IC_{50}\right)}$$

The combination neutralization curve is then calculated using the Bliss Independence model,

f=1−(1−f(A))(1−f(B))…(1−f(N))

with f(A), f(B), etc. being the individual functions of the IgG antibodies. Combination IC_50_ or IC_80_ titers are calculated by setting f = 0.5 or 0.8 and assuming each IgG is present at the same concentration.

Active dual or triple coverage was calculated by considering that respectively both or all three antibodies were able to neutralize the virus at a given concentration.

### Generation of logograms for the V1V2 region

The nucleotide alignment of the clade C viruses, containing the reference sequence for HXB2 were translated to amino acid space, using the LANL tool “Codon Alignment v2.1.0” (https://www.hiv.lanl.gov/content/sequence/CodonAlign/codonalign.html). Logograms were created for the viruses from the following groups: 1998–2006 (n = 96), 2007–2010 (n = 104), 2016–2020 Placebo (n = 31) using the LANL tool “Analyze Align” (https://www.hiv.lanl.gov/content/sequence/ANALYZEALIGN/analyze_align.html). Sites included in the logogram were colour-coded according to available information on resistance/sensitivity to neutralization by CAP256-VRC26.25 [22]. The sites were numbered according to HXB2 position.

### Generation of Heatmaps

IC80 neutralization data from the two trials was used to generate heatmaps and clustering analysis. Heatmaps were created using the LANL tool “Heatmap” (https://www.hiv.lanl.gov/content/sequence/HEATMAP/heatmap_mainpage.html) with the option of hierarchical clustering. Clusters were compared using Fisher exact tests.

### Variable loop characteristics

The same data that were used as input for generating the logograms, were used to analyze variable loop characteristics. Characteristics of the variable loop regions V1, V2, V1V2, V3, V4 and V5 were analyzed using the LANL tool “Variable Region Characteristics” (https://www.hiv.lanl.gov/cgi-bin/VAR_REG_CHAR). Results were imported into R for analysis.

### Statistical analysis

Comparisons between groups were done using the Mann-Whitney U test on GraphPad Prism. This was applied to the neutralization sensitivities of viruses in the placebo arm compared to those in the VRC01 arm of the AMP trial. A Jonckheere-Terpstra test was used to compare the IC80s of all AMP placebo viruses to the 200 Clade C Panel viruses using the package “DescTools” [54]. A Dunn test with Bonferroni correction for multiple testing was also used to determine which pair(s) of periods had significant differences. P values of <0.05 were considered statistically significant. Statistical analyses were generated using R (version 4.1.2 (2021-11-01) — "Bird Hippie") (R Core Team, 2021) and graphs plotted using GraphPad Prism.

## Supporting information

S1 Table Neutralization profiles of viruses from the AMP trials assayed against bnAbs in clinical development. (XLSX)

S2 Table Predicted coverage of bnAb combinations against circulating viruses. (XLSX)
